# *dupA*^+^ *H. pylor*i reduces diversity of gastric microbiome and increases risk of erosive gastritis

**DOI:** 10.3389/fcimb.2023.1103909

**Published:** 2023-03-17

**Authors:** Ruiyan Chen, Ying Li, Xiaodong Chen, Jianhui Chen, Jie Song, Xiaoqiao Yang, Lifang Ye, Zizhong Wu, Peng Xie, Qiong Zhong, Runshi Yang, Jiachuan Wu

**Affiliations:** ^1^Digestive Endoscopy Center, Guangdong Second Provincial General Hospital, Guangzhou, China; ^2^Guangdong Provincial Key Laboratory of Microbial Safety and Health, State Key Laboratory of Applied Microbiology Southern China, Institute of Microbiology, Guangdong Academy of Sciences, Guangzhou, China; ^3^Division of Gastrointestinal Surgery Center, The First Affiliated Hospital of Sun Yat-Sen University, Guangzhou, China; ^4^Department of Gastroenterology, Longnan Hospital of Traditional Chinese Medicine, Longnan, China

**Keywords:** Helicobacter pylori, dupA, gastric microbiome, erosive gastritis, microbial diversity

## Abstract

Helicobacter pylori is believed to induce gastropathy; however, the exact pathogenic molecules involved in this process have not been elucidated. Duodenal ulcer promoting gene A (DupA) is a virulence factor with a controversial role in gastric inflammation and carcinogenesis. To explore and confirm the function of DupA in gastropathy from the perspective of the microbiome, we investigated the microbial characteristics of 48 gastritis patients through 16S rRNA amplicon sequencing. In addition, we isolated 21 H. pylori strains from these patients and confirmed the expression of dupA using PCR and qRT-PCR. Bioinformatics analysis identified diversity loss and compositional changes as the key features of precancerous lesions in the stomach, and H. pylori was a characteristic microbe present in the stomach of the gastritis patients. Co-occurrence analysis revealed that H. pylori infection inhibits growth of other gastric inhabiting microbes, which weakened the degradation of xenobiotics. Further analysis showed that dupA+ H. pylori were absent in precancerous lesions and were more likely to appear in erosive gastritis, whereas dupA− H. pylori was highly abundant in precancerous lesions. The presence of dupA in H. pylori caused less disturbance to the gastric microbiome, maintaining the relatively richness of gastric microbiome. Overall, our findings suggest that high dupA expression in H. pylori is correlated with a high risk of erosive gastritis and a lower level of disturbance to the gastric microbiome, indicating that DupA should be considered a risk factor of erosive gastritis rather than gastric cancer.

## 1 Introduction

*Helicobacter pylori* is a gram-negative, microaerophilic, helix-shaped bacterium that is believed to induce gastritis, gastric ulcers, and even cancer (Suerbaum and Michetti, 2002; Baj et al., 2020). Epidemiological investigations showed that *H. pylori* infects approximately 30–80% of people worldwide and poses a great threat to human health (Ren et al., 2022; Sonnenberg, 2022). Therefore, research on the invasive mechanism of *H. pylori* is crucial for its prevention and treatment.

Despite its high prevalence, only 1–3% of *H. pylori*-infected patients develop gastric cancer, indicating that not all *H. pylori* strains are carcinogenic (Peek and Crabtree, 2006). Recent research has revealed that the carcinogenesis and invasiveness of *H. pylori* are associated with its virulence factors (Baj et al., 2020). These virulence factors interact with the gastric epithelium and alter the microenvironment of the stomach, thereby enhancing carcinoma development (Noto et al., 2019; Zhao et al., 2019). Among these virulence factors, the most widely studied are cytotoxin-associated gene A and vacuolating cytotoxin A (Ferreira et al., 2016; Maeda et al., 2017, Raghwan and Chowdhury, 2014). These two toxins can activate mucosal inflammation and induce cellular proliferation, which can convert normal mucosal cells into cancer cells (McClain et al., 2017; Chauhan et al., 2019; Alipour, 2021). However, not all virulence factors of *H. pylori* are directly associated with carcinogenesis, and their influence on gastric mucosa requires further exploration.

Duodenal ulcer promoting gene A (DupA) is a molecule responsible for mucosal inflammation in the stomach (Lu et al., 2005; de Lima Silva et al., 2021). However, the influence of DupA on gastric carcinogenesis remains controversial (Abadi et al., 2012; Alam et al., 2012; de Lima Silva et al., 2021). Takahashi et al. reported that *dupA*-positive (*dupA*^+^) *H. pylori* strains contribute to gastric cancer *via* induction of gastritis (Takahashi et al., 2013). In contrast, Imagawa performed a retrospective cohort study and found that *dupA* expression was negatively associated with gastric cancer (Imagawa et al., 2010). Therefore, further investigation of the relationship between *dupA* expression and gastric carcinogenesis is needed to explain the exact function of this virulence factor.

To elucidate the effects of microbiome on gastropathy, we explored changes in the gastric microbial communities caused by gastritis and precancerous lesions using *16S rRNA* amplicon sequencing and identified the key carcinogenic microbial functions and their contributors. In addition, we evaluated the influence of *dupA*^+^ *H. pylori* strains on gastropathy and elucidated *dupA* expression by the gastric microbiome to delineate the influence of DupA on gastric diseases from a microbiome perspective.

## 2 Materials and methods

### 2.1 Study cohort and sample collection

A total of 48 gastric mucosa biopsy tissues were collected from the First Affiliated Hospital of Sun Yat-sen University from June 1 to July 1, 2021. Of these, 34 tissue samples were collected from patients with superficial gastritis (SG), 9 from those with erosive gastritis (EG), 3 from those with atrophic gastritis (AG), and 2 from those with intestinal metaplasia (IM). All samples were obtained from the antrum through endoscopic examination. The inclusion criteria for participants in this study were as follows: over 18 years old and under 65 years old; no history of malignant diseases, cardiopulmonary diseases, metabolic diseases, or autoimmune diseases; no history of gastrectomy or reflux esophagitis; no history of *H. pylori* eradiation, antibiotics, proton pump inhibitor, or H_2_ receptor antagonist treatment within the past 3 months. Written informed consent was obtained from all patients, and this study was conducted according to the guidelines of the Declaration of Helsinki and approved by the ethics committee of the First Affiliated Hospital of Sun Yat-sen University (project number 2020-164).

The mucosal samples from each patient were divided into two pieces aseptically; one was stored in the Sample Protector (TAKARA, Beijing, China) and transferred to −80 °C until DNA extraction, while the other one was stored in the *H. pylori* protection fluid (Huankai Biology, Guangzhou, Guangdong, China) and transferred to the laboratory for *H. pylori* isolation.

### 2.2 *H. pylori* isolation

*H. pylori* strains were isolated from the mucosal tissues according to the method as previously described (Li et al., 2022). In brief, the mucosal samples were inoculated on a *H. pylori* selective agar (Huankai Biology) at 37 °C in a microaerophilic incubator (Binder, Tuttlingen, Germany) containing 10% O_2_, 5% CO_2_, and 85% N_2_ for up to 10 days. The small gray and translucent colonies were picked and purified on a new *H. pylori* selective agar for genomic DNA extraction. The taxonomic analysis of the *H. pylori*-like colonies was performed using *16S rRNA* sequencing with the universal primers (27F: 5′-AGAGTTTGATCCTGGCTCAG-3′; 1492R: 5′-ACGGCTACCTTGTTACGACTT-3′) (Garrity, 2016). In all, 18 strains of *H. pylori* were isolated from the 48 patients in this study.

### 2.3 PCR amplification

The PCR amplification of *dupA* of the *H. pylori* strains was carried out according to the method described by Xue using the *dupA* primer (*dupA−*F: 5′-GACGATTGAGCGATGGGAATAT-3′; *dupA−*R: 5′- CTGAGAAGCCTTATTATCTTGTTGG-3′) (Xue et al., 2021). The genomic DNA of *H. pylori* was extracted using the microbial DNA extraction kit (Huankai Biology). PCR amplification was carried out in a 25-μL reaction mix containing 0.2 μM forward and reverse primers, 50 ng genomic DNA template, 12.5 μL PrimeSTAR Max DNA Polymerase (TAKARA), and nuclease-free water. Samples were denatured at 98 °C for 1 min, followed by 35 cycles at 98 °C for 10 s, 55 °C for 15 s, and 72 °C for 5 s, with a final elongation at 72 °C for 30 s. The amplified products were analyzed by electrophoresis at 120 V for 25 min in a 1.5% agarose gel containing 1 × Tris-acetate-EDTA (TAE) buffer, stained with GoldView Staining Dyes (Solarbio, Beijing, China), and visualized using a gel documentation system (Bio-Rad, Hercules, CA, USA). Samples showing a monoclonal brand of 971 bp were considered as *dupA*^+^ *H. pylori* strains.

### 2.4 *dupA* expression assay by qRT-PCR

The expression of *dupA* was examined according to the method of Alam (Alam et al., 2012). Total RNA of *H. pylori* was extracted by TRIzol reagent according to the manufacturer’s protocol (TAKARA). cDNA was generated using the Master Mix cDNA Synthesis Kit (Accurate Biotechnology, Changsha, Hunan, China). Quantitative reverse transcription PCR (qRT-PCR) of *dupA* was performed using SYBR Green I (Accurate Biotechnology) using the primers of DupAsetIF/DupAsetIR, with *rpsT* being the reference (**Table 1**). The mean Ct of triplicate reactions was determined and the expression levels of *dupA* were measured using the -ΔCt method. The relative expression of dupA in H. pylori isolates was calculated as 2^-ΔΔCt^, where -ΔΔCt =-ΔCt_sample_ - ΔCt_reference_. *H. pylori* strain P6, which was isolated from the SG patient, was selected as the reference strain in the qRT-PCR assay.

**Table 1:** Primers used in this study for qRT-PCR.

Primers	Sequence (5’→3’)	Amplicon (bp)	Annealing temp (°C)
DupAsetI			
Forward	CGTGATCAATATGGATGCTT	214	54
Reverse	GCAAAGTGTTCCGTTGATCT		
RpsT			
Forward	GGCAAATCATAAGTCCGCAGAA	217	55
Reverse	CTTTCCTAGAAGCGGTGTTTTTCT		

### 2.5 Mucosal microbial DNA extraction and *16S rRNA* amplicon sequencing

Microbial DNA of the gastric mucosa was extracted using the QIAamp PowerFecal Pro DNA kit (Qiagen, Hilden, Germany). *16S rRNA* gene was amplified using the V3–V4 hypervariable region primers 338F 5′-ACTCCTACGGGAGGCAGCAG-3′ and 806R 5′-GGACTACHVGGGTWTCTAAT-3′, with the amplicon length of 468 bp (Fadrosh et al., 2014). PCR was performed using the UCP Multiplex PCR Kit (Qiagen), which involved denaturation at 95 °C for 2 min, followed by 30 cycles at 95 °C for 5 s, 55 °C for 15 s, and 72 °C for 30 s, with a final elongation at 72 °C for 30 s. The amplicon libraries for next-generation sequencing were generated using the QIAseq Ultralow Input Library Kit (Qiagen).

The amplicon libraries were validated and pooled by the Agilent high sensitivity DNA kit (Agilent, Santa Clara, CA, USA). Paired-end sequencing was conducted on the MiSeq platform with the MiSeq Reagent Kit version V3 (Illumina, San Diego, CA, USA).

### 2.6 Bioinformatic analysis of the gastric mucosal microbiome

Quality filtering of the data was performed using the CLC Genomic Workbench version 20.0 (Qiagen). The trimmed sequences were matched to those in the Greengenes database (v13.5) with similarity cutoff of 97% and clustered into operational taxonomic units (OTUs).

Taxonomic analysis was performed using the Microbiome Analyst software (Chong et al., 2020). Microbial diversity was explored based on the Chao1 and Shannon indices at the genus level using analysis of variance (ANOVA). In addition, microbial composition was investigated using non-metric multidimensional scaling (NMDS) at the genus level among different groups. Linear discriminant analysis effect size (LEfSe) was used to determine the key taxonomic differences among groups, with the threshold on the logarithmic linear discriminant analysis score of 2.0 for discriminative features and alpha value of 0.05 for the pairwise Wilcoxon test (Segata et al., 2011).

Metagenome prediction of the mucosal microbiome was performed using the Phylogenetic Investigation of Communities by Reconstruction of Unobserved States (PICRUSt) (Langille et al., 2013). The statistical differences between the groups were examined in the second and third levels of the Kyoto Encyclopedia of Genes and Genomes (KEGG) pathway analysis using ANOVA. Correlation analysis was conducted between the key genera and metabolic pathways in the gastric mucosa using Pearson’s correlation test.

Network analysis was performed to identify the co-occurring relationship using Pearson’s correlation test, and results were visualized using Cytoscape v3.9.0 (Shannon et al., 2003). The core genera in gastric microbiome were represented as nodes (red for key genera in AG/IM, blue for key genera in EG, grey for genera in all groups), relative abundance (RA) as node size, and edges depicted correlations (green for positive correlation, grey for correlation without significance).

## 3 Results

### 3.1 Basic characteristics of the gastric microbiome in gastritis patients

The clinical characteristics of the study cohort are summarized in **Table 2**. No significant differences were found in age and gender among the SG, EG, and AG/IM patients.

**Table 2:** Clinical characteristics of the study cohort.

	SG (n = 34)	EG (n = 9)	AG/IM (n = 5)	*P*
**Age** (yrs) (median, range)	45 (20 ~ 63)	49 (29 ~ 58)	53 (28 ~ 60)	0.398
Gender				
Male (cases) (%)	15 (44.12)	5 (55.55)	4 (80.00)	0.304
Female (cases) (%)	19 (55.89)	4 (44.44)	1 (20.00)	
***H. pylori* isolation** (case) (%)	12 (35.29)	6 (66.67)	3 (60.00)	0.187
***dupA* + *H. pylori* infection** (case) (%)	1 (2.94)	5 (55.56)	0 (0.00)	0.002

SG, Superficial gastritis; EG, Erosive gastritis; AG/IM, Atrophic gastritis/Intestinal metaplasia.

In this study, 972 OTUs were observed in the gastric mucosa of 48 patients with gastritis. These gastric microbes were distributed into 11 phyla, 19 classes, 35 orders, 65 families, and 87 genera. The most commonly detected phyla in the gastric microbiome were *Proteobacteria* (all samples), *Firmicutes* (89.58% of samples), *Bacteroidetes* (81.25% of samples), *Actinobacteria* (9.17% of samples), and *Fusobacteria* (77.08% of samples). The most commonly detected genera in the gastric microbiome were *Streptococcus* (83.33% of samples), *Prevotella* (81.25% of samples), *Neisseria* (75.00% of samples), *Fusobacterium* (75.00% of samples), *Veillonella* (70.83% of samples), and *Rothia* (70.83% of samples).

### 3.2 Gastric microbiota profile in SG, EG, and AG/IM

**Figure 1:**
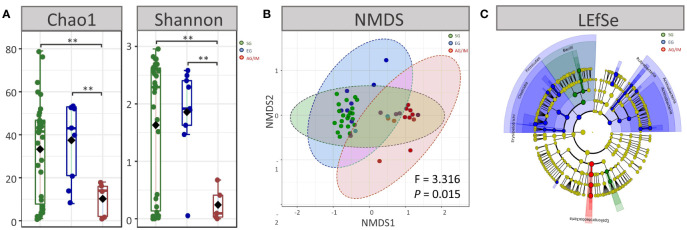
Microbial profiles of SG, EG, and AG/IM patients. **(A)** Alpha diversity at genus level in superficial gastritis (SG), erosive gastritis (EG), and atrophic gastritis/intestinal metaplasia (AG/IM) patients. **(B)** Beta diversity at genus level in SG, EG and, AG/IM patients. **(C)** Colored cladogram of linear discriminant analysis effect size analysis showing microbes with biomarker significance in gastric mucosa of SG, EG, and AG/IM patients (green for biomarkers in SG, blue for biomarkers in EG, and red for biomarkers in AG/IM). **P < 0.01 between groups.

The microbial diversity of each sample was evaluated at the genus level based on Chao1 and Shannon indices. A comparison of alpha diversity revealed that the highest richness of microbes was observed in the mucosa of SG patients, while the lowest was observed in AG/IM patients (*P* < 0.001 for both Chao1 and Shannon indices) (**Figure 1A**).

Moreover, we found that the microbial composition of the mucosa differed among SG, EG, and AG/IM patients (*P* = 0.016). The gastric microbial composition of AG/IM patients was different from that of SG patients (PERMANOVA, R^2^ = 0.371, *P* = 0.011) and EG patients (PERMANOVA, R^2^ = 0.140, *P* = 0.002) based on the Bray–Curtis index. However, no difference in the microbial composition of the mucosa was observed between the SG and EG patients (PERMANOVA, R^2^ = 0.010, *P* = 0.736) (**Figure 1B**).

To identify the dominant microbes in different patients with gastritis, LEfSe analysis was performed. The results showed that *Pasteurellaceae* and *Gemellaceae* were the most prevalent families in SG patients, whereas *Prevotellaceae*, *Veillonellaceae*, *Clostridiaceae*, *Actinomycetaceae*, and *Rubrobacteraceae* were common in EG patients, and *Helicobacteraceae* was the most prevalent family in AG/IM patients (**Figure 1C**).

### 3.3 Microbial function profile in SG, EG, and AG/IM

**Figure 2:**
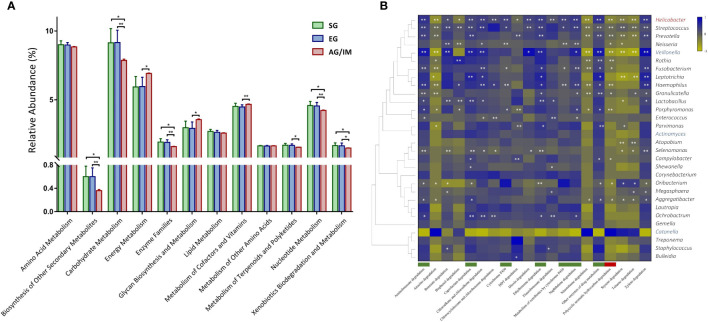
Microbial function profile in SG, EG, and AG/IM patients. **(A)** Comparison of microbial function of the gastric microbiome in superficial gastritis (SG), erosive gastritis (EG), and atrophic gastritis/intestinal metaplasia (AG/IM) in 12 metabolic pathways related to microbial metabolism. **(B)** Correlation analysis between the core gastric microbes and key metabolic pathways in xenobiotic biodegradation shown as a heatmap based on Pearson’s correlation analysis. The Pearson’s correlation coefficient between the genus and metabolic pathway was calculated and is shown as a colored matrix (blue represents a positive correlation, while yellow represents a negative correlation). Thirty core genera were arranged according to their taxonomic evolution, with the key genera in EG marked in blue, the key genus in AG/IM marked in red, and the other taxa marked in black. Downregulated and upregulated metabolite functions in AG/IM are marked in green and red, respectively. Statistical significance is expressed as *P < 0.05 and **P < 0.01.

Overall, 5739 microbial metabolites in 276 metabolic pathways were identified by PICRUSt in the gastric microbiome in this study. Among them, a higher number of genes involved in cellular processes and fewer genes involved in microbial metabolism were detected in the gastric microbes of AG/IM patients compared to the SG and EG patients (*P* = 0.042 and *P* = 0.026, respectively). As microbial metabolites might exert carcinogenic effects on epithelial cells (Imai et al., 2021; Singhal et al., 2021), we further analyzed the changes in the 12 metabolic pathways in patients with AG/IM. Our results showed that the microbial activities of carbohydrate metabolism, enzyme families, nucleotide metabolism, biosynthesis of other secondary metabolites, and xenobiotic biodegradation and metabolism were significantly lower in the gastric microbes of AG/IM patients, whereas the activities of energy metabolism, glycan biosynthesis, and metabolism of cofactors and vitamins were higher (*P* < 0.05) (**Figure 2A**).

We further analyzed the functional changes of 20 xenobiotics in the gastric microbiome and found that the microbial activities of drug metabolism associated with cytochrome P450 and other enzymes, ethylbenzene degradation, aminobenzoate degradation, naphthalene degradation, and caprolactam degradation were significantly decreased in AG/IM patients (*P* < 0.05), while the microbial activities associated with polycyclic aromatic hydrocarbon degradation increased (*P* = 0.013). Correlation analysis revealed that microbial dysfunction was related to changes in key microbes in the gastric mucosa (**Figure 2B**).

### 3.4 *H. pylori* infection of the study cohort

**Figure 3:**
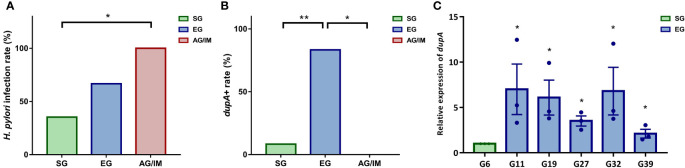
*H pylori* infection rate in the study cohort. **(A)** *H pylori* isolation rate in superficial gastritis (SG), erosive gastritis (EG), and atrophic gastritis/intestinal metaplasia (AG/IM) patients. **(B)** *dupA*^+^ rate in the *H pylori* strains isolated from SG, EG, and AG/IM patients. **(C)** Relative expression of *dupA* in *H pylori* strains isolated from SG and EG patients. * means *P* < 0.05 compared to reference strain. *P < 0.05 between groups or compared to the reference strain and **P < 0.01 between groups.

Although OTUs of *H. pylori* were found in 23 patients based on amplicon sequencing, only 21 *H. pylori* strains were isolated from mucosal samples. Of these, 12 strains were isolated from patients with SG, six from those with EG, and three from those with AG/IM (**Figure 3A**).

PCR analysis revealed that among these *H. pylori* isolates, six strains were *dupA*^+^ and 15 were *dupA^−^*. Further investigation revealed that 83.33% of the *dupA*^+^ *H. pylori* strains were isolated from the mucosal samples of the EG patients and 16.67% were isolated from the SG patients, indicating that the infection rate of the *dupA*^+^ *H. pylori* strain was higher in EG patients than in both SG and AG/IM patients (*P* = 0.002) (**Figure 3B**). qRT-PCR of the six *dupA^+^* strains revealed that the relative level of *dupA* transcript was higher in the EG isolated strains than the SG isolated strains (*P* = 0.002) (**Figure 3C**).

### 3.5 Microbial characteristics of *dupA*^+^ *H. pylori* infection

DupA is an *H. pylori* virulence factor that increases the risk of mucosal ulcers (Queiroz et al., 2011). As all *dupA*^+^ *H. pylori* strains were found in SG and EG patients, we further investigated the influence of these strains on the gastric microbiome in these patients.

**Figure 4:**
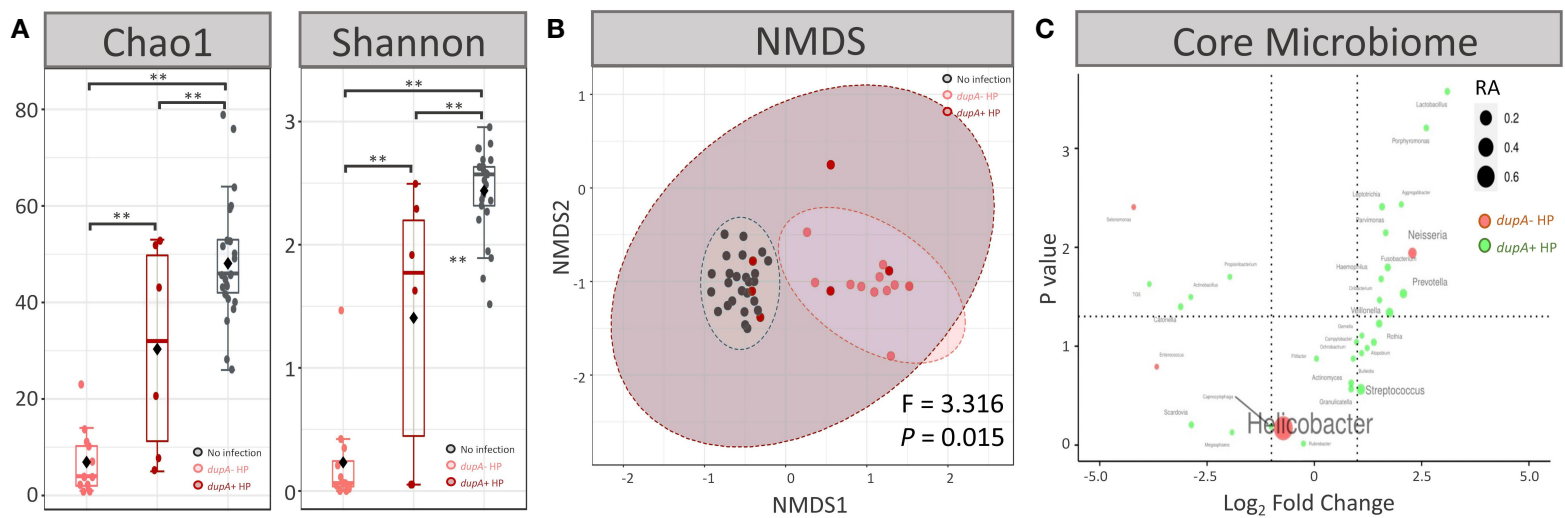
Microbial characteristics of *dupA*^+^ *H pylori*-infected patients. **(A)** Alpha diversity at genus level in *dupA*^+^ *H pylori*-infected patients and *dupA*^–^ *H pylori*-infected patients. **(B)** Beta diversity at genus level in *dupA*^+^ *H pylori*-infected patients and *dupA*^–^ *H pylori*-infected patients. **(C)** Volcano plot of the key genus in the gastric microbiome. Comparisons of the relative abundances (RA) of each genus shown as log_2_ fold change on the X-axis and *P*-value on the Y-axis. The average RA of each genus is shown as node size and genera with higher RAs in *dupA*^+^ *H pylori*-infected patients are shown in green, whereas genera with higher RAs in *dupA*^–^ *H pylori*-infected patients are shown in green. **P < 0.01 between groups.

Microbial diversity analysis revealed that genus richness decreased after *dupA*^+^ *H. pylori* infection (*P* < 0.01 for both Chao1 and Shannon indices), and similar changes occurred in *dupA^−^ H. pylori* infection (*P* < 0.01 for both Chao1 and Shannon indices) (**Figure 4A**). Further investigation showed that *dupA^−^ H. pylori* infection induced a more severe loss of biodiversity than *dupA*^+^ *H. pylori* infection (*P* = 0.041 for the Chao1 index and *P* = 0.044 for Shannon index) (**Figure 4A**). Moreover, the microbial composition changed as a result of both *dupA*^+^ *H. pylori* and *dupA*^−^ *H. pylori* infection (*P* < 0.01 for both groups compared with the uninfected group in NMDS analysis). Differences in microbial composition were also observed in *dupA*^+^ *H. pylori*- and *dupA*^−^ *H. pylori*-infected patients (*P* = 0.015) (**Figure 4B**).

ANOVA revealed that the RA of *Leptotrichia*, *Bulleidia*, *Capnocytophaga*, *Gemella*, *Lactobacillus*, *Aggregatibacter*, *Atopobium*, and *Ochrobactrum* was higher in *dupA*^+^ *H. pylori*-infected patients than in *dupA^−^ H. pylori*-infected patients (*P* < 0.05) (**Figure 4C**).

### 3.6 Alteration of gastric microbiome in *dupA*^+^ *H. pylori* infection

**Figure 5:**
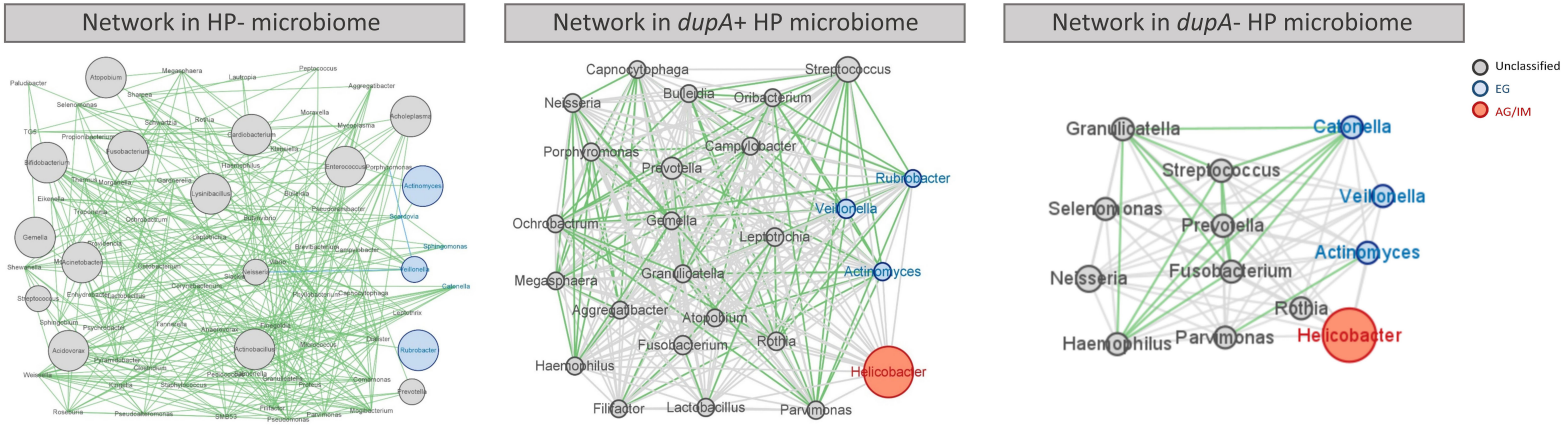
Co-occurrence network of the gastric microbes of different *H. pylori*-infected patients. Co-occurrence of gastric bacteria at the genus level in *H. pylori*-infected patients. Genera are presented as nodes (EG specific genera in blue and AG/IM group-specific genera in red), genus abundance is presented as node size, and edges are based on the associations detected using Pearson’s correlation analysis (positive inter-node correlations in green, correlations without statistical significance in grey).

As the commensal microbes in the human body compete and cooperate with each other and act as important players in maintaining our health (Faust et al., 2012), further exploration of the gastric microbial network might help to better understand the influence of *H. pylori* infection on the gastric mucosa. In this study, we observed a complex correlation network among the key genera in mucosal microbes without *H. pylori* infection, most of which were in a mutually beneficial relationship (**Figure 5A**). Infection with *dupA*^+^ *H. pylori* resulted in reduced microbial diversity, weakened interactions between microbes, and the disappearance of mutual promotion between probiotic bacteria such as Streptococcus and Prevotella (**Figures 5A, B**). Most significantly altered gastric microbiome was observed in *dupA*^−^ *H. pylori*-infected patients, with a loss of most probiotics in the gastric microbiome (**Figure 5C**).

## 4 Discussion

The advent of high-throughput sequencing has expanded our understanding of the effect of the human microbiome on health status (Consortium, 2019, Dominguez-Bello et al., 2019). Owing to its high acidity, a healthy stomach has long been regarded as a sterile organ, and the presence of microbes such as *H. pylori* induces inflammation and even cancer (Mégraud et al., 2016). However, previous studies have shown that a variety of microorganisms inhabit a healthy stomach and that these microbes have a great influence on the homeostasis of the gastric mucosa (Ianiro et al., 2015; Rajilic-Stojanovic et al., 2020). Therefore, exploring the gastric microbiome and its interactions with epithelial cells may help improve our knowledge on the maintenance of gastric health.

In this study, we found that the gastric mucosa of patients with gastritis was inhabited by a variety of microbes, such as *Proteobacteria* and *Firmicutes*. This finding is consistent with those of Parsons et al. and Ferreira et al. (Parsons et al., 2017; Ferreira et al., 2018), indicating that the composition of the gastric microbiome is relatively stable among different populations with differing lifestyles. For a long time, *H. pylori* was thought to be the only bacterium capable of surviving in the acidic environment of the stomach (Stingl et al., 2002); however, our results revealed that *Streptococcus* and *Prevotella* were the most common inhabiting microbes in the gastric mucosa, regardless of the presence of *H. pylori*. Therefore, it is reasonable to deduce that gastric microorganisms other than *H. pylori* may also exert carcinogenic effects on epithelial cells.

To test our hypothesis, we analyzed the changes in gastric microbial profiles in case of SG, EG, and AG/IM. Our results revealed that microbial diversity and composition differed among various types of mucosal lesions, and the low diversity and high RA of *Helicobacter* were the key features of AG/IM. In addition, we noticed that the RA of *Pasteurellaceae* and *Gemellaceae* was higher in SG, while the RA of *Prevotellaceae*, *Veillonellaceae*, *Clostridiaceae*, *Actinomycetaceae*, and *Rubrobacteraceae* was higher in EG (*P* < 0.05). Differences in the microbial composition of the gastric mucosa might lead to apparent microbial interactions with the gastric mucosa (Coker et al., 2018; Liu et al., 2019; Noto et al., 2019). Therefore, we further compared the microbial functional characteristics of SG, EG, and AG/IM. Our results showed that the microorganisms in AG/IM had the weakest microbial metabolic function. Further analysis revealed that the microbes in AG/IM possessed lower activities in degrading xenobiotics, such as ethylbenzene, naphthalene, and caprolactam (*P* < 0.05). As the carcinogenic effects of these chemicals have been well-proven (Swaen et al., 2005; Bailey and Rhomberg, 2020; Khorrami et al., 2021), failure to degrade them in the stomach may increase the risk of gastric cancer. Further correlation analysis showed that core microbes such as *Streptococcus*, *Prevotella*, and *Lactobacillus* were the key contributors to xenobiotic degradation, and loss of these microbes in AG/IM might induce precancerous lesions in the gastric mucosa.

*Helicobacter* was identified as the key pathogen in AG/IM. As *H. pylori* is the only species in the genus that can infect humans, we isolated *H. pylori* strains from the gastric mucosa and analyzed the influence of their virulence factors on the gastric epithelium. As DupA is a newly discovered virulence factor in *H. pylori* and its influence on gastric inflammation as well as cancer remains controversial (Gomes et al., 2008, Abadi and Perez-Perez, 2016), we examined its existence and expression in different groups of patients and its pathogenic function with respect to the microbiome. We discovered that although no differences in the *H. pylori* isolation rates were found among SG, EG and AG/IM, the *dupA*^+^ strains were more common in the EG group. These results indicated that *dupA*^+^ *H. pylori* strains were associated with severe inflammation and ulcers, while *dupA^−^ H. pylori* strains were more prone to inducing precancerous lesions in the stomach. Furthermore, higher expression of *dupA* was found in the EG isolated *H. pylori* strains, indicating that DupA is a risk factor of gastric ulcer. Our findings are consistent with those of a clinical cohort study and suggest that DupA may not be a risk factor of gastric carcinogenesis (Hussein, 2010; Alam et al., 2020).

To further explore the interactions between DupA and the gastric mucosa, we compared the gastric microbial profiles of *dupA*^+^ and *dupA*^−^ *H. pylori*-infected patients, as well as patients without *H. pylori* infection. Our results demonstrated that both *dupA*^+^ and *dupA*^−^ *H. pylori* infection reduced microbial diversity and changed the microbial composition in the gastric microbiome (*P* < 0.01), and these effects were more pronounced in patients with *dupA*^−^ *H. pylori* infection (*P* < 0.05). Compared to *dupA*^−^ *H. pylori* infection, *dupA*^+^ *H. pylori* infection caused fewer changes in the prevalence of core microbes in the stomach. In addition, co-occurrence network analysis revealed that *H. pylori* infection disrupted the integrity of the microbial interaction network in the stomach and caused the disappearance of mutually beneficial relationships among the core gastric microbes. Compared to *dupA*^−^ *H. pylori* infection, *dupA*^+^ *H. pylori* infection induced fewer changes in the gastric microbiome, and the core microbes that produce metabolites beneficial to the mucosa could promote each other’s growth in the *dupA*^+^ *H. pylori*-infected microbiome. These findings indicate that *dupA*^+^ *H. pylori* causes fewer disturbances in the homeostasis of the gastric microbiome, resulting in a lower level of carcinogenic metabolites in the gastric mucosa. The revealed microbial influence of DupA was contrary to that of CagA, which is a well-known carcinogenic protein of *H. pylori* (Noto et al., 2019). These findings indicated that the existence and expression of DupA of *H. pylori* should be monitored in gastritis patients with severe inflammatory and ulcers; however, no evidence was found in this study to support its association with precancerous lesions. However, the role of DupA in gastritis and its influence on gastric microbiome requires verification in a larger clinical cohort, as well as experiments on cellular and animal models, which will help to provide more precise diagnostic markers for clinical diagnosis.

In conclusion, our study explored the microbial characteristics of SG from EG to AG/IM and showed that *H. pylori* is the key pathogen in gastric precancerous lesions. Functional microbial analysis revealed that *H. pylori* inhibited the growth of other microbes inhabiting the gastric mucosa and weakened the degradation of carcinogenic xenobiotic metabolites. Furthermore, the influence of *H. pylori* on the gastric microbiome was found to be related to its virulence factor DupA. The *dupA* expression in *H. pylori* decreased the disturbance of mucosal homeostasis and increased the risk of erosive gastritis.

## Data availability statement

The datasets presented in this study can be found in online repositories. The names of the repository/repositories and accession number(s) can be found below: https://www.ncbi.nlm.nih.gov/genbank/PRJNA903497.

## Ethics statement

The studies involving human participants were reviewed and approved by the ethics committee of the First Affiliated Hospital of Sun Yat-sen University. The patients/participants provided their written informed consent to participate in this study.

## Author contributions

Conceptualization, JW, RC, and YL. methodology, RC and JW. Bioinformatic analysis, YL, JS, and QZ. project administration, RC, YL, XC, XY, LY, and RY. resources, JC. data investigation, ZW and PX. writing—original draft preparation, RC. writing—review and editing, YL and JW. funding acquisition, YL and RY. All authors contributed to the article and approved the submitted version.

## References

[B1] Abadi A. T. B. Perez-Perez G. (2016). Role of *dupA* in virulence of *Helicobacter pylori* . World J. Gastroenterol. 22, 10118–10123. doi: 10.3748/wjg.v22.i46.10118 28028359 PMC5155170

[B2] Abadi A. T. B. Taghvaei T. Wolfram L. Kusters J. G. (2012). Infection with *Helicobacter pylori* strains lacking *dupA* is associated with an increased risk of gastric ulcer and gastric cancer development. J. Med. Microbiol. 61, 23–30. doi: 10.1099/jmm.0.027052-0 21903829

[B3] Alam J. Maiti S. Ghosh P. De R. Chowdhury A. Das S.. (2012). Significant association of the *dupA* gene of *Helicobacter pylori* with duodenal ulcer development in a south-east Indian population. J. Med. Microbiol. 61, 1295–1302. doi: 10.1099/jmm.0.038398-0 22653921

[B4] Alam J. Sarkar A. Karmakar B. C. Ganguly M. Paul S. Mukhopadhyay A. K. (2020). Novel virulence factor *dupA* of *Helicobacter pylori* as an important risk determinant for disease manifestation: An overview. World J. Gastroenterol. 26, 4739–4752. doi: 10.3748/wjg.v26.i32.4739 32921954 PMC7459207

[B5] Alipour M. (2021). Molecular mechanism of *Helicobacter pylori*-induced gastric cancer. J. Gastrointestinal Cancer 52, 23–30. doi: 10.1007/s12029-020-00518-5 32926335 PMC7487264

[B6] Bailey L. A. Rhomberg L. R. (2020). Incorporating ToxCast™ data into naphthalene human health risk assessment. Toxicol. Vitro 67, 104913. doi: 10.1016/j.tiv.2020.104913 32526344

[B7] Baj J. Forma A. Sitarz M. Portincasa P. Garruti G. Krasowska D.. (2020). *Helicobacter pylori* virulence factors-mechanisms of bacterial pathogenicity in the gastric microenvironment. Cells 10, 27. doi: 10.3390/cells10010027 33375694 PMC7824444

[B8] Chauhan N. Tay A. C. Y. Marshall B. J. Jain U. (2019). *Helicobacter pylori* vacA, a distinct toxin exerts diverse functionalities in numerous cells: An overview. Helicobacter 24, e12544. doi: 10.1111/hel.12544 30324717

[B9] Chong J. Liu P. Zhou G. Xia J. (2020). Using MicrobiomeAnalyst for comprehensive statistical, functional, and meta-analysis of microbiome data. Nat. Protoc. 15, 799–821. doi: 10.1038/s41596-019-0264-1 31942082

[B10] Coker O. O. Dai Z. Nie Y. Zhao G. Cao L. Nakatsu G.. (2018). Mucosal microbiome dysbiosis in gastric carcinogenesis. Gut 67, 1024–1032. doi: 10.1136/gutjnl-2017-314281 28765474 PMC5969346

[B11] Consortium (2019). The integrative human microbiome project. Nature 569, 641–648. doi: 10.1038/s41586-019-1238-8 31142853 PMC6784865

[B12] de Lima Silva L. L. Oliveira A. K. S. Gama A. R. Ramos A. Silva A. Blanco A. J. V.. (2021). *Helicobacter pylori* virulence *dupA* gene: Risk factor or protective factor? Brazilian journal of microbiology Braz J Microbiol 52, 1921–1927. doi: 10.1007/s42770-021-00553-9 34255308 PMC8578514

[B13] Dominguez-Bello M. G. Godoy-Vitorino F. Knight R. Blaser M. J. (2019). Role of the microbiome in human development. Gut 68, 1108–1114. doi: 10.1136/gutjnl-2018-317503 30670574 PMC6580755

[B14] Fadrosh D. W. Ma B. Gajer P. Sengamalay N. Ott S. Brotman R. M.. (2014). An improved dual-indexing approach for multiplexed *16S rRNA* gene sequencing on the illumina MiSeq platform. Microbiome 2, 6. doi: 10.1186/2049-2618-2-6 24558975 PMC3940169

[B15] Faust K. Sathirapongsasuti J. F. Izard J. Segata N. Gevers D. Raes J.. (2012). Microbial co-occurrence relationships in the human microbiome. PloS Comput. Biol. 8, e1002606. doi: 10.1371/journal.pcbi.1002606 22807668 PMC3395616

[B16] Ferreira R. M. Pereira-Marques J. Pinto-Ribeiro I. Costa J. L. Carneiro F. Machado J. C.. (2018). Gastric microbial community profiling reveals a dysbiotic cancer-associated microbiota. Gut 67, 226–236. doi: 10.1136/gutjnl-2017-314205 29102920 PMC5868293

[B17] Ferreira R. M. Pinto-Ribeiro I. Wen X. Marcos-Pinto R. Dinis-Ribeiro M. Carneiro F.. (2016). *Helicobacter pylori* cagA promoter region sequences influence CagA expression and interleukin 8 secretion. J. Infect. Dis. 213, 669–673. doi: 10.1093/infdis/jiv467 26401027

[B18] Garrity G. M. (2016). A new genomics-driven taxonomy of bacteria and archaea: Are we there yet? J. Clin. Microbiol. 54, 1956–1963. doi: 10.1128/JCM.00200-16 27194687 PMC4963521

[B19] Gomes L. I. Rocha G. A. Rocha A. M. Soares T. F. Oliveira C. A. Bittencourt P. F.. (2008). Lack of association between *Helicobacter pylori* infection with *dupA*-positive strains and gastroduodenal diseases in Brazilian patients. Int. J. Med. Microbiol. IJMM 298, 223–230. doi: 10.1016/j.ijmm.2007.05.006 17897881

[B20] Hussein N. R. (2010). The association of *dupA* and *Helicobacter pylori*-related gastroduodenal diseases. Eur. J. Clin. Microbiol. Infect. Dis. 29, 817–821. doi: 10.1007/s10096-010-0933-z 20419465

[B21] Ianiro G. Molina-Infante J. Gasbarrini A. (2015). Gastric microbiota. Helicobacter 20 (Suppl 1), 68–71. doi: 10.1111/hel.12260 26372828

[B22] Imagawa S. Ito M. Yoshihara M. Eguchi H. Tanaka S. Chayama K. (2010). *Helicobacter pylori dupA* and gastric acid secretion are negatively associated with gastric cancer development. J. Med. Microbiol. 59, 1484–1489. doi: 10.1099/jmm.0.021816-0 20829397

[B23] Imai S. Ooki T. Murata-Kamiya N. Komura D. Tahmina K. Wu W.. (2021). Helicobacter pylori CagA elicits BRCAness to induce genome instability that may underlie bacterial gastric carcinogenesis. Cell Host Microbe 29, 941–958.e910. doi: 10.1016/j.chom.2021.04.006 33989515

[B24] Khorrami Z. Pourkhosravani M. Rezapour M. Etemad K. Taghavi-Shahri S. M. Künzli N.. (2021). Multiple air pollutant exposure and lung cancer in Tehran, Iran. Sci. Rep. 11, 9239. doi: 10.1038/s41598-021-88643-4 33927268 PMC8085005

[B25] Langille M. G. Zaneveld J. Caporaso J. G. McDonald D. Knights D. Reyes J. A.. (2013). Predictive functional profiling of microbial communities using 16S rRNA marker gene sequences. Nat. Biotechnol. 31, 814–821. doi: 10.1038/nbt.2676 23975157 PMC3819121

[B26] Li Y. Huang Z. Shang Y. Xie X. Yang R. Chen H.. (2022). Exploration of the molecular mechanisms underlying the antibiotic resistance of helicobacter pylori: A whole-genome sequencing-based study in southern China. Helicobacter 6, e12879. doi: 10.1111/hel.12879 35124867

[B27] Liu X. Shao L. Liu X. Ji F. Mei Y. Cheng Y.. (2019). Alterations of gastric mucosal microbiota across different stomach microhabitats in a cohort of 276 patients with gastric cancer. EBioMedicine 40, 336–348. doi: 10.1016/j.ebiom.2018.12.034 30584008 PMC6412016

[B28] Lu H. Hsu P. I. Graham D. Y. Yamaoka Y. (2005). Duodenal ulcer promoting gene of *Helicobacter pylori* . Gastroenterology 128, 833–848. doi: 10.1053/j.gastro.2005.01.009 15825067 PMC3130061

[B29] Maeda M. Moro H. Ushijima T. (2017). Mechanisms for the induction of gastric cancer by *Helicobacter pylori* infection: aberrant DNA methylation pathway. Gastric Cancer 20, 8–15. doi: 10.1007/s10120-016-0650-0 27718135

[B30] McClain M. S. Beckett A. C. Cover T. L. (2017). *Helicobacter pylori* vacuolating toxin and gastric cancer. Toxins 9, 316. doi: 10.3390/toxins9100316 29023421 PMC5666363

[B31] Mégraud F. Lehours P. Vale F. F. (2016). The history of *Helicobacter pylori*: from phylogeography to paleomicrobiology. Clin. Microbiol. infection 22, 922–927. doi: 10.1016/j.cmi.2016.07.013 27451940

[B32] Noto J. M. Zackular J. P. Varga M. G. Delgado A. Romero-Gallo J. Scholz M. B.. (2019). Modification of the gastric mucosal microbiota by a strain-specific *Helicobacter pylori* oncoprotein and carcinogenic histologic phenotype. mBio 10, e00955–e00919. doi: 10.1128/mBio.00955-19 31138752 PMC6538789

[B33] Parsons B. N. Ijaz U. Z. D'Amore R. Burkitt M. D. Eccles R. Lenzi L.. (2017). Comparison of the human gastric microbiota in hypochlorhydric states arising as a result of helicobacter pylori-induced atrophic gastritis, autoimmune atrophic gastritis and proton pump inhibitor use. PloS Pathog. 13, e1006653. doi: 10.1371/journal.ppat.1006653 29095917 PMC5667734

[B34] Peek R. M. Jr. Crabtree J. E. (2006). *Helicobacter* infection and gastric neoplasia. J. Pathol. 208, 233–248. doi: 10.1002/path.1868 16362989

[B35] Queiroz D. M. Rocha G. A. Rocha A. M. Moura S. B. Saraiva I. E. Gomes L. I.. (2011). dupA polymorphisms and risk of *Helicobacter pylori*-associated diseases. Int. J. Med. Microbiol. IJMM 301, 225–228. doi: 10.1016/j.ijmm.2010.08.019 21050811

[B36] Raghwan Chowdhury R. (2014). Host cell contact induces fur-dependent expression of virulence factors CagA and VacA in *Helicobacter pylori* . Helicobacter 19, 17–25. doi: 10.1111/hel.12087 24020886

[B37] Rajilic-Stojanovic M. Figueiredo C. Smet A. Hansen R. Kupcinskas J. Rokkas T.. (2020). Systematic review: Gastric microbiota in health and disease. Aliment Pharmacol. Ther. 51, 582–602. doi: 10.1111/apt.15650 32056247

[B38] Ren S. Cai P. Liu Y. Wang T. Zhang Y. Li Q.. (2022). Prevalence of *Helicobacter pylori* infection in China: A systematic review and meta-analysis. J. Gastroenterol. Hepatol. 37, 464–470. doi: 10.1111/jgh.15751 34862656

[B39] Segata N. Izard J. Waldron L. Gevers D. Miropolsky L. Garrett W. S.. (2011). Metagenomic biomarker discovery and explanation. Genome Biol. 12, R60. doi: 10.1186/gb-2011-12-6-r60 21702898 PMC3218848

[B40] Shannon P. Markiel A. Ozier O. Baliga N. S. Wang J. T. Ramage D.. (2003). Cytoscape: A software environment for integrated models of biomolecular interaction networks. Genome Res. 13, 2498–2504. doi: 10.1101/gr.1239303 14597658 PMC403769

[B41] Singhal R. Donde H. Ghare S. Stocke K. Zhang J. Vadhanam M.. (2021). Decrease in acetyl-CoA pathway utilizing butyrate-producing bacteria is a key pathogenic feature of alcohol-induced functional gut microbial dysbiosis and development of liver disease in mice. Gut Microbes 13, 1946367. doi: 10.1080/19490976.2021.1946367 34369304 PMC8354657

[B42] Sonnenberg A. (2022). Epidemiology of *Helicobacter pylori* . Aliment Pharmacol. Ther. 55 Suppl 1, S1–13. doi: 10.1111/apt.16592 34989430

[B43] Stingl K. Altendorf K. Bakker E. P. (2002). Acid survival of *Helicobacter pylori*: How does urease activity trigger cytoplasmic pH homeostasis? Trends Microbiol. 10, 70–74. doi: 10.1016/S0966-842X(01)02287-9 11827807

[B44] Suerbaum S. Michetti P. (2002). *Helicobacter pylori* infection. N Engl. J. Med. 347, 1175–1186. doi: 10.1056/NEJMra020542 12374879

[B45] Swaen G. M. Scheffers T. de Cock J. Slangen J. Drooge H. (2005). Leukemia risk in caprolactam workers exposed to benzene. Ann. Epidemiol. 15, 21–28. doi: 10.1016/j.annepidem.2004.03.007 15571990

[B46] Takahashi A. Shiota S. Matsunari O. Watada M. Suzuki R. Nakachi S.. (2013). Intact long-type dupA as a marker for gastroduodenal diseases in okinawan subpopulation, Japan. Helicobacter 18, 66–72. doi: 10.1111/j.1523-5378.2012.00994.x PMC3545078 23067336

[B47] Xue Z. Yang H. Su D. Song X. Deng X. Yu C.. (2021). Geographic distribution of the cagA, vacA, iceA, oipA and dupA genes of *Helicobacter pylori* strains isolated in China. Gut Pathog. 13, 39. doi: 10.1186/s13099-021-00434-4 34130751 PMC8207754

[B48] Zhao Y. Gao X. Guo J. Yu D. Xiao Y. Wang H.. (2019). Helicobacter pylori infection alters gastric and tongue coating microbial communities. Helicobacter 24, e12567. doi: 10.1111/hel.12567 30734438 PMC6593728

